# The Relationship between the COVID-19 Pandemic and Nursing Students’ Sense of Belonging: The Experiences and Nursing Education Management of Pre-Service Nursing Professionals

**DOI:** 10.3390/ijerph17165848

**Published:** 2020-08-12

**Authors:** Luis Miguel Dos Santos

**Affiliations:** Woosong Language Institute, Woosong University, Daejeon 34514, Korea; luismigueldossantos@yahoo.com; Tel.: +82-010-3066-7818

**Keywords:** COVID-19 pandemic, nursing education, nursing student, pre-service nursing professional, public health development, sense of belonging, social distancing

## Abstract

The COVID-19 pandemic has changed the orders and structures of societies, particularly in the fields of medical and nursing professions. The researcher aims to understand the experiences, sense of belonging, and decision-making processes about Japanese pre-service nursing students and how the COVID-19 pandemic, social distancing, and lockdown has influenced their understanding as pre-service nursing professionals in Japan. As this study focuses on the issues of pre-service nursing students, the researcher invited forty-nine pre-service nursing students for a virtual interview due to the recommendation of social distancing. To increase the coverage of the population, the researcher employed snowball sampling to recruit participants from all over Japan. Although the COVID-19 pandemic influenced the overall performance of the medical and nursing professions, all participants showed a sense of belonging as Japanese citizens and nursing professionals due to the natural disaster of their country. More importantly, all expressed their desires and missions to upgrade and improve the overall performance of the public health system due to the influence of the COVID-19 pandemic. The results discovered that many Japanese nursing students advocated that Japan’s national development, the benefits and advantages of their country, were of a greater importance than their own personal development and goals.

## 1. Introduction

### 1.1. Purpose of the Study

Nursing training and education is not a liberal arts study, but rather a vocational-oriented training for pre-service nursing professionals at the university level. Although nursing students may spend time on campus for theoretical courses and general education requirements, a large portion of their time should be spent in clinical internships and placements [1]. However, due to the international COVID-19 pandemic, many face-to-face courses and internships in clinical environments are affected by social distancing recommendations. Although the COVID-19 pandemic does not change the enrolment status and registration procedure of pre-service nursing students, their experiences, sense of belonging, and decision-making processes must be influenced by external and environmental problems, particularly the COVID-19 pandemic. 

This study has three purposes. First, the researcher aims to understand the experiences, sense of belonging, and career decision-making processes [2,3,4,5] of nursing students from two time periods (i.e., before the COVID-19 pandemic and during the COVID-19 pandemic). It is important to understand how the COVID-19 pandemic influences and impacts the behaviours of nursing students in order to ensure effective human resource management and school enrolment plans.

Second, due to the COVID-19 pandemic, recommendations of social distancing, and distance learning-based teaching and learning experiences, some students may defer their studies or drop their university studies altogether. Due to the COVID-19 pandemic, many nursing students may not be able to complete their internships and placements at the clinical level due to the lockdown governmental policy. Therefore, the researcher would like to understand how these elements influence the experiences, sense of belonging, and decision-making processes [2,3,4,5] of a group of nursing students in Japan.

Finally, with a focus on the sense of belonging, the researcher seeks to understand how the relationship between a sense of patriotism as Japanese citizens and the COVID-19 pandemic influence the experiences and decision-making processes of a group of nursing students in Japan [6,7].

In short, the current research study was guided by one research question:

1. Based on the lens of the social cognitive career theory, what are the motivations and reasons that influenced the experiences, sense of belonging, and career decision-making processes of nursing students in Japan? How would you describe?

The interview questions and focuses on these aspects—and the results and discussion are divided into two time periods (i.e., before the COVID-19 pandemic and during the COVID-19 pandemic)—in order to outline the differences between public health conditions.

### 1.2. Literature Review

In general, nursing occupations are stable and come with a higher social status, but are also characterised by work and responsibility overloads, usually causing their practitioners to be tired, and are low-paying in order to broaden opportunities across occupational disciplines for those entering the workforce [8]. Although work–life responsibilities are not balanced due to human resource shortages, it is important to increase the overall enrolment and experience of nursing students and in-service nursing professionals in order to ensure adequate workforce management [9]. A previous study [2,10] indicated that a large number of mid-level and senior-level nursing professionals decided to leave the profession due to poor administrative management, overloaded working responsibilities, insufficient salaries, and regular overtime. However, many hospital leaders, clinical managers, policymakers, and government leaders do not have solid and tailor-made human resource plans for this particular workforce (i.e., nursing professionals), as general human resources planning may not be able to respond to the needs of the medical and nursing areas [11].

Besides ineffective human resources management, recruitment in the medical and nursing profession is not the same as in other nonprofit and profit-making industries. Based on the current nonprofit management scheme, many human resources professionals advocate that the medical and nursing profession may share significant elements and factors with education, social care, and psychology professions [10,12]. Although these fields are generally considered to be nonprofit, medical and nursing professionals may start their own business-oriented clinics and hospitals for profit-making purposes. Based on these studies, it is worth noting that the job nature and working environment may not match prospective students’ expectations, experiences, sense of belonging, and decision-making processes, as members of the general public generally believe nursing to be a nonprofit profession. Such unbalanced expectations may cause confusion in pre-service nursing students and junior-level nursing professionals [13,14].

The nature of jobs in Japan is another consideration for individuals’ experiences, their sense of belonging, and decision-making processes in the field of nursing [6]. Unlike many other countries, Japanese people tend to stay in the same company for life-long career development. Although some working environments do not match their expectations, Japanese people always stay in the same organisation once they have graduated from school. An early study [15] explored the relationship between gender, job responsibilities, positions and roles of female health and medical professionals in Japan based on their socioeconomic backgrounds, qualifications, career developments and patients’ perspectives. The female participants indicated that the public health sector and the medical profession is their life-long career. They would spend their energies and contribute to the field and their organisations without questioning. More importantly, the results also indicated that the gender differences between males and females might create a social bias due to their gender. However, they could adjust their mindset in order to provide excellent services to their patients. Another recent study [16] indicated that after Japan has a mandatory retraining programme which allowed and encouraged nursing professionals to return to the same organisation after any career breaks and issues. In other words, resigned nursing professionals should have the right to come back to the same nursing position or the same organisation after some situations, such as becoming a mother. As a result, Japanese people always investigate the background, nature, performance, feedback, and reputation of organisations before committing themselves to a life-long career [17].

### 1.3. Theoretical Framework

Experiences, sense of belonging, and decision-making processes are not single direction elements, but multiple factors which can interconnect with each other [18]. In this study, the researcher tended to understand the experiences, sense of belonging and decision-making processes of Japanese nursing students during the COVID-19 pandemic and how the COVID-19 pandemic influenced their behaviours. As a result, the researcher decided to employ the social cognitive career theory (SCCT) [19,20,21,22,23,24,25] as the tool to explore and investigate the abovementioned elements, the performance and the limitation in individuals’ education and career goals [26,27,28,29,30].

The development of the SCCT [1,2,3,4,5,6,7,8,9,10,11,12,13,14] was based on the foundation of social cognitive theory [31,32], with the additional conceptions of career decision, development, and perspective, focusing on the individual’s understanding, behaviour, financial consideration, and external/environmental factors. Both Bandura [31,32] and Lent et al. [24,25] believed that individuals’ behaviours and thinking should not be a direction and single element but multiple connections and interactions [33]. These multiple behaviours and activities can be directions to interact and connect individuals’ career perspectives and understanding [7].

First, human behaviours and decision-making processes are not in a single direction, they go in multiple directions, ways and conducts. Career-related interest is one of the factors influencing individuals’ career decisions. For example, a previous study [29] indicated that female scientists and engineers decided to switch to science, technology, engineering and mathematics (STEM) education due to their personal goals and career interests in training and teaching. Individual career decisions may be changed due to the various elements and situations. The SCCT, therefore, provides the tool to explore this direction.

Second, academic and career achievements also serve as a consideration in career development. For example, a recent study [7] explored the reasons and motivations of why male nursing professionals decided to switch their career development to nursing education. The results indicated that male nursing educators believed their academic and career experiences would be beneficial to the next generation. Therefore, the motivations of these groups of participants were mainly focused on their academic and career achievements for their qualifications and goals.

Third, performance and persistence in educational and occupational pursuits is another significant element for career development and career decisions. A recent study [30] indicated that the connections between educational and occupational pursuits would influence individuals’ career development and career decisions. For example, nursing professionals who face a high level of stress and burnout may leave their position and the profession due to the psychological distress and low-level of satisfaction in their profession [34]. As a result, individuals’ behaviours and career perspectives are impacted by thinking, internal elements, emotions, external/environmental factors, and even financial considerations collectively [29].

To illustrate the SCCT and the related concepts, the SCCT has three important points for its modelling in the notions and directions of career decision and development [24,25,33].

- First, the formation and elaboration of career-related interest.
- Second, the election of academic and career selection and direction options.
- Third, the performance and persistence in educational and occupational pursuits.

**Figure 1:**
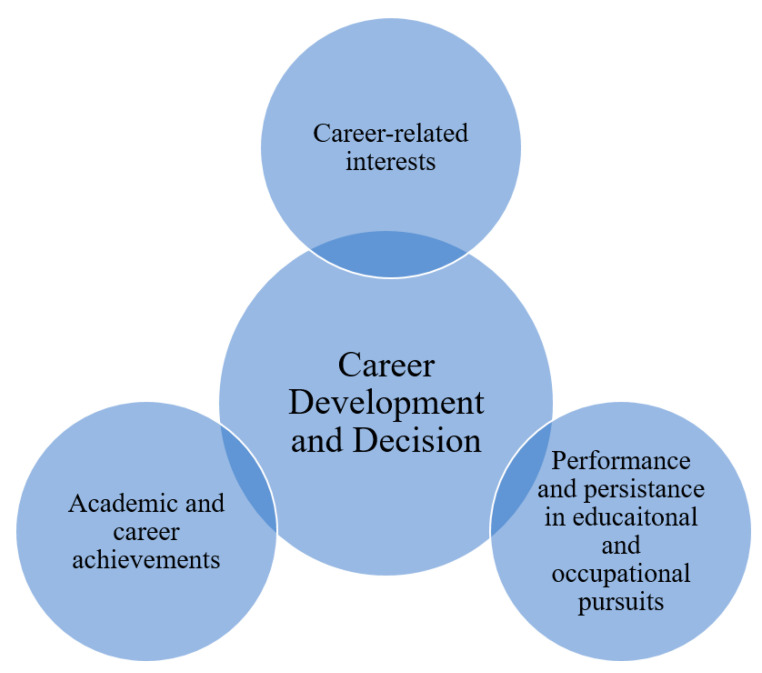
The social cognitive career connections between career development and decision.

SCCT [1,2,3,4,5,6,7,8,9,10,11,12,13,14] argues that the theory has categorised the differences and directions between personal intentions and wants (i.e., personal beliefs, personal goals and purposes, internal desires, dreams) and behaviours (i.e., activities, movements, actions, conducts, ways and decision-making processes), as individuals tended to base their conduct on what they usually believe and advocate. In other words, individuals tend to conduct and practice their career decisions and selection based on what they advocate instead of human resources management and practices from other individuals, groups, and government leadership [1,2,3,4,5,6,7,8,9,10,11,12,13,14]. To illustrate, please refer to Figure 1.

## 2. Materials and Methods

As an established qualitative researcher [26,27,28,35,36,37,38,39] in the field of public health, education, nursing, and social sciences, the researcher decided to employ a qualitative research method to collect and analyse the data information into meaningful themes and subthemes for reporting. The nature of this study is to understand and explore the lived stories, shared information, life experiences, sense of belonging, and decision-making processes [33,40,41,42,43,44] of nursing students during the COVID-19 pandemic in Japan.

### 2.1. Participants and Recruitment

As of April 2017, according to the Japan Nursing Association [45], there were 277 public health nursing schools and nearly 22,000 active nursing students in Japan. Over 95% of these nursing students were enrolled at one of the nursing schools at the senior-level colleges and universities for at least a bachelor’s degree qualification.

A total of forty-nine Japanese nursing students were invited. All agreed to participate in this study. The snowball sampling strategy [38,46] was employed to recruit the participants. First, based on personal networking and connections, the researcher was able to invite 11 nursing students who could meet the criteria of the research study. Second, the participants were encouraged to forward the research protocol to other potential nursing students with a similar background. Third, after several rounds of invitations and referral activities, a total of forty-nine participants joined the study. After the researcher believed the meaningful data information and shared information had met the saturation (i.e., similar shared information and feedback were repeated without additional themes and ideas), the researcher decided to suspend the recommendations of referral activities.

First, some scholars may make arguments about the numbers of participants in this study (i.e., the numbers are not enough). According to Moustakas [47], qualitative research studies tend to focus on interpersonal communication, the sharing of lived stories and the quality of the data information. The quality of the data information is more important than the quantity of the numbers. Clandnin and Connelly [41] also suggested that qualitative researchers should focus on intensive sharing and an in-depth understanding of the participants in order to collect the real and true data from the shared information of the participants.

Second, in order to increase the rich and meaningful data information of the qualitative research studies, Merriam [46] suggested that qualitative research studies should recruit at least three participants and no more than 100 participants. Moustakas [47] further suggested that a standard phenomenological analysis should recruit no more than 50 participants for a high-level qualitative study. Therefore, based on the guidelines from various qualitative researchers, the current research study met the expectations as a qualitative research study.

Due to the concerns of privacy, particularly in the field of medical and nursing professions with limited networks and connections, the researcher needed to assign a pseudonym to each participant in order to mask their identity to potential supervisors, clinical sites, internship and placement managers, government agencies, and policymakers. Therefore, the official name, university name, place of origin, and specialisation have been masked as these elements did not impact the results of the study. The demography of the participants has been listed within the Appendix Table A1 section.

### 2.2. Data Collection

As this research study was conducted during the COVID-19 pandemic, the government and the World Health Organisation (WHO, Geneva, Switzerland) had established the social distancing recommendation. Therefore, face-to-face interview sessions were not encouraged. As a result, the researcher could only conduct the interview sessions via social media virtual chats (i.e., WhatsApp and Line Chat).

The open-ended and semi-structured interview tools [38,39,46] were employed to collect meaningful data and information from the participants. The individuals shared their understanding, life experiences, sense of belonging, decision-making processes, and feedback as Japanese nursing students [33,40,41,42,43,44] and how the COVID-19 pandemic influenced their behaviours. All participants have voluntarily participated in this study. The general inductive approach [48] was employed for the qualitative data collection and analysis. Appendix B lists the interview questions. It is worth noting that the interview questions were developed based on the recommendations and guidelines of SCCT [1,2,3,4,5,6,7,8,9,10,11,12,13,14], recent studies [6,7], and the objectives of the study.

In order to seek rich, informative data from the participants, the researcher first sent a detailed academic and personal background to the participants. According to Seidman [49,50], a relationship between the researcher and participants should be established in order to collect in-depth data information and sharing. However, due to the COVID-19 pandemic and the related limitations, such as the social distancing recommendation, the in-depth interview sessions [38,46] and closed relationships could not be established. However, in order to establish a virtual relationship between each other, first, the researcher sent detailed information to each for review. Second, before the interview session, each received the interview questions at least ten days before the virtual chat. Third, the participants could contact the researcher for any academic recommendations and suggestions, on topics such as homework tuition or career development. Although not all participants contacted the researcher prior to the formal interview session(s), the researcher tried his best to increase the interactions between each other during this COVID-19 pandemic.

As for the virtual interview sessions, each interview session lasted from 58 to 78 min. Before the interview sessions started, the researcher explained the rights of participation, risks, and benefit to each participant [38,46]. Additionally, the researcher used an audio recorder to record the interview sessions for further data analysis. All orally agreed and finished the interview sessions without interruption. In addition to the interview sessions, after the researcher finished the data collection and analysis procedures, the researcher sent the related materials to each participant for confirmation and member checking. All agreed with their collected data and confirmed the validity [35].

None of the participants were native English speakers but Japanese speakers. However, all had a superior level of English language skills with solid third-language backgrounds (e.g., Chinese Mandarin, Russian, Arabic, Korean, French, German, and Spanish). All were informed that they could use both English and Japanese for the interview sessions. However, all decided to use English as the language for sharing.

### 2.3. Data Analysis

After the data collection procedure, the oral conversation has been transcribed to written transcripts for reporting. Qualitative researchers [26,27,28,35,36,37,38,39] advocated that large-size data information should be narrowed down to systematic themes and groups for reporting. Therefore, the researcher employed the open-coping strategy [6,7,33,38,46] to narrow down the data information to the first-level themes and subthemes. As a result, 23 themes and 34 subthemes were categorised.

However, it is not recommended to have more than ten themes and ten subthemes for a manuscript. Therefore, the researcher continued to employ the axial-coding strategy [38,46] to narrow down the first-level themes and subthemes into second-level themes and subthemes [6,7,33,38,46]. As a result, two themes and four subthemes were categorised. Please refer to Table 1 for the themes and subthemes. 

### 2.4. Human Subjects Protection

All of the signed and unsigned agreements and content forms, personal contacts, audio recordings, written transcripts, computers, and related materials were locked in a password-protected cabinet. Only the researcher has the rights to read the materials. After the study was completed, the researcher immediately destroyed and deleted the materials for personal privacy.

Due to the content forms and agreements, the official names, university names, places of origin, and specialisations have been masked as these elements did not impact the results of the study. However, the participants agreed that their university location and current grade could be shown. All subjects gave their informed consent for inclusion before they participated in the study. The study was conducted in accordance with the Declaration of Helsinki, and the protocol was approved by the University Ethics Committee (2020/02/03).

### 2.5. Validity of the Qualitative Data Information

The validity of the qualitative research data information is important. Therefore, the researcher tried his best to exercise some procedures in order to increase the rate of validity. According to Robson [51], qualitative researchers should exercise at least one solution to ensure the validity of qualitative research studies. In this study, the researcher exercised three ways to increase validity. First, the prolonged involvement, although the researcher could not see the participants in private due to the recommendation of social distancing. However, the researcher provided the tutorial sessions and practices as the tool to increase the involvement between each other.

Second, as mentioned above, the researcher asked the participants for the member checking procedure. The researcher sent the related materials to each participant for confirmation and member checking. All agreed with their data information and confirmed the validity.

Third, the audit trail was used in this study as well. The researcher kept all the related materials and data information in his diary and notebooks. Each step was recorded and marked with detailed information and notices.

After the researcher exercised these three procedures for the validity of the data information, the current research results and findings should meet the recommendation on how to confirm the qualitative research data information [46,51].

## 3. Results and Discussion

During the interview sessions, the participants answered the same general semi-structured questions, which aimed to capture their personal lived stories, understanding, concepts, perspectives, and opinions about their intentions and motivations (i.e., to become a nurse in the future). Although their places of origin, living environments, family backgrounds, educational histories, and personal expectations were not the same, many shared a similar understanding and feedback about their motivations and reasons for pursuing nursing education and training, as well as their decision-making process (i.e., becoming a nurse).

According to SCCT [19,20,21,22,23,24,25], people’s experiences, sense of belonging, and decision-making processes might be different due to the current international COVID-19 pandemic; an internal understanding and external/environmental factors [19,20,21,22,23,24,25] can highly influence the overall understanding, behaviours, and decision-making processes of individuals. In this case, in order to measure the differences between the period before and after the COVID-19 pandemic, the researcher categorised the themes and subthemes based on two different timeframes (i.e., before the COVID-19 pandemic and during the COVID-19 pandemic). In fact, as this research study was conducted during the COVID-19 pandemic, the researcher could only capture data for the second timeframe (i.e., during the COVID-19 pandemic).

Based on the data, none of the participants changed their mind regarding becoming a registered nurse after university graduation due to the COVID-19 pandemic and related influences. Although the COVID-19 pandemic may damage some government policies, benefits packages, job responsibilities, expectations, and even working hours, none of the participants decided to quit their dream job as they all had solid purposes and personal goals. Table 1 outlines the themes and subthemes of this study. 

### 3.1. Before the COVID-19 Pandemic: I Have the Mission to Help Minorities

The original aim of this study was to explore the experiences, sense of belonging, and decision-making processes [19,20,21,22,23,24,25] of university nursing students in Japan. However, as the COVID-19 pandemic has influenced the global order, public health systems, and the understanding and feedback of professionals in the public health system, it is meaningful to understand how professionals in the public health system describe their experiences, sense of belonging, and decision-making processes [19,20,21,22,23,24,25]. In this case, the researcher invited forty-nine university nursing students in Japan as the sample. The results from this study indicated that these factors were not highly influenced by the recent COVID-19 pandemic.

By listening to the sharing and life experiences [35,41] of the participants, the researcher identified several meaningful factors that impacted the decision-making process. The findings of this study revealed that all of the participants will complete their nursing degree, will try their best to reach a higher grade for better skills, and will join the nursing profession in rural, remote, and suburban regions for minorities, such as senior citizens. By employing the lens of SCCT [19,20,21,22,23,24,25], the researcher discovered that all the participants had decided to enrol in the nursing education programme due to their sense of patriotism [52] and their desire to promote public health for minorities [53] in their country. As one participant said:

“Due to the urban-oriented city developments, most of the youths and secondary graduates tend to go to large cities and regions for career development, such as Tokyo, Osaka, and Kyoto … so, many senior citizens stay in the rural communities without help … as a Japanese citizen, I have the mission to help minorities, particularly all people in rural communities … they cannot be ignored …”

(P#27,Chubu, Junior)

#### 3.1.1. Promoting Rural and Suburban Public Health Performance and Knowledge

All forty-nine participants expressed that there was room for improvement in the current Japanese public health system. Although Japan is one of the most developed countries in the East Asian region, its public health coverage and doctor–patient ratio are lacking. The large group of participants was originally from and studying in rural regions and communities. Therefore, many of them had a mission to serve rural residents. A previous study [54] indicated that public health professionals tended to leave rural communities due to the limitations of career promotions and salary differences. However, based on the data information from this study, the participants expressed the interests and goals of rural health promotion.

At the personal level [19,20,21,22,23,24,25], different participants expressed various ideas about how being in a nursing programme and potential nursing position would allow them to increase the quality of the public health system and a sense of personal belonging [19,20,21,22,23,24,25] in order to use their skills and knowledge to upgrade the aged health systems and facilities for both mental and physical satisfaction [55,56,57]. For example, one participant advocated that his/her nursing knowledge could help the rural community health system in upgrading:

“not only are the health facilities in the rural regions old, the doctors and nurses are old as well … they will retire in a decade … if we do not want to improve the rural community health care system, how can we improve the overall experience of our citizens? If some people don’t want to go back, I will … and I need, this is my mission …”

(P#35, Hokkaido, Senior)

In fact, not just one participant shared this idea. Almost all participants shared similar ideas about rural health system improvement. As one participant said:

“all regions in Japan need doctors, nurses, and therapists, not just the urban cities … my purpose after graduation is to increase and improve our rural health system in my home province… I love my country and I love my people … this is not only about myself and my own wish, Japanese people have the mission to serve the country …”

(P#44, Kansai, Senior)

Another participant also indicated that the rural communities in Japan are facing significant human resources shortages due to urbanisation and the decreasing population in rural communities [58]. Therefore, if there are no medical and nursing professionals entering the rural communities in the coming decade, a large number of citizens, particularly senior citizens, will lose their basic caring services, as one said:

“I would like to conduct my placement in the rural community if possible … the health system and practice in urban and rural hospitals are not the same … I like to take care of senior citizens as they served our country in their younger years … as a young Japanese citizen, I can see I have the mission to take care of them in later life …”

(P#21, Tohoku, Junior)

Based on SCCT [19,20,21,22,23,24,25], many of the participants stated that their sense of belonging (i.e., patriotism, the mission of nursing professionals, and feeling for their hometown) always encouraged their experiences and decision-making processes. No one mentioned financial considerations or lack of career promotion opportunities [12] due to the limited resources in rural regions [58]. When compared with some previous studies, this group of participants seems to have had a more solid understanding of and firmer ideas about their own selections and choices. One of the significant findings was the sense of belonging for the development of rural communities. Unlike the results from a previous study [54], the results of this study advocated the interests of career decisions and career development in rural communities. This finding highlighted the potential workforce gaps problems that might be solved.

#### 3.1.2. Increasing the Quality of the Public Health System as a Citizen

According to a previous study [52], besides concerns regarding rural health system development, many advocated that, as Japanese citizens, they have their own missions and responsibilities to contribute their knowledge and energy to the public health system. More than 40 participants mentioned that Japan had faced a significantly low birth rate for more than two decades [59]. Without positive public health promotion, female health promotion, family caring, and sexual health promotion, the effective increase in quality cannot be supported [60]. The ideas about contribution to a country are uncommon for many public health studies, as most of the studies focused on the ideas about personal interests, career developments and education for the next generation [6,7,61]. Therefore, the following sharing might highlight the unique behaviours and career decision-making process for Japanese nursing students.

Within this subtheme, the researcher categorised the connection between two aspects of their sense of belonging, namely as Japanese citizens and as nursing professionals, as a whole group for reporting. Under this category, many participants shared interests in and goals regarding the promotion of the public health system as Japanese citizens. One said:

“we need to have a stronger public health care system … the current Japanese public health system is still very junior. There is much room for improvement … but many medical leaders and government agencies do not understand the thinking and behaviours of our people … the youths, adults, and even senior citizens’ health problems … as a nursing student, I have my mission to take care of the promotion …”

(P#4, Kanto, Freshman)

Another participant advocated that female health and sexual health should be promoted in order to establish a better environment, particularly regarding pregnancy and births:

“I want to reform the sexual promotion health system for female Japanese people … many female individuals do not understand how to protect their body. I have the mission and responsibility to promote females’ health rights and concerns for a better environment … if we do not promote and protect females’ rights, no female individuals will give birth to babies …”

(P#18, Shikoku, Sophomore)

Based on SCCT [19,20,21,22,23,24,25], many participants explained that their interest in becoming a nurse was due to their sense of vocation and sense of belonging (i.e., as Japanese citizens) [52]. Unlike previous studies [1,2,10,62,63,64], in which nursing students selected their career development based on personal goals and financial considerations, in this case, many expressed the mission of being Japanese citizens [52]. Several messages were captured. Two significant messages were captured:

“the natural resources in Japan are not strong … due to the inequality in the remote communities, many need to move to the urban or suburban environments … therefore, the government started to spend many resources on the rural communities … although these are the facts, we still cannot neglect the needs of poor residents in urban environments, such as the homeless people in Osaka … Japanese people need to help Japanese people …”

(P#49, Okinawa, Senior)

“Not only the rural communities, but all the patients and minorities in the urban and rural communities … different people need different services … but health promotion is always needed … I always discuss the health promotion plans and topics with my supervisors and professors … I have to promote these topics after I graduate, as a citizen …”

(P#41, Chubu, Senior)

It is worth noting that many Japanese people have very strong sense of patriotism [52]. Based on SCCT [19,20,21,22,23,24,25], many advocated that their sense of belonging as Japanese citizens and as nursing professionals was their motivation for studying nursing as their university major and making their career investment in the nursing profession [6]. Many believed that the development of Japan was much more important than their own personal goals and interests [27,65,66]. The significant findings from these themes and subthemes highlighted the unique sense of belonging and career decision-making process of Japanese nursing students. The ideas of collectivism [67] and country (i.e., Japan) dominated many aspects of the behaviours [52]. These aspects, however, led to the sense of belonging and career decision-making processes of these groups of participants.

### 3.2. During the COVID-19 Pandemic: My Country Needs My Effort and Energy

Under the interview questions and protocol, the researcher gathered the original motivations for becoming nursing professionals and how the COVID-19 pandemic influenced the experiences, sense of belonging, and decision-making processes of a group of Japanese nursing students through the lens of SCCT [19,20,21,22,23,24,25]. Under this theme, the researcher reported the participants’ shared information and life experiences regarding how COVID-19 changed their ideas.

However, after the data analysis procedure, the researcher discovered that the COVID-19 pandemic has reinforced rather than changed their experiences, sense of belonging, and decision-making processes. Through the lens of SCCT [19,20,21,22,23,24,25], many expressed that their thinking, ideas, and understanding (i.e., of becoming a nursing professional after graduation) have been strengthened. Based on the sense of belonging and collectivism [67], Japanese nursing students expressed interests in serving their country and hopeless patients. These senses and behaviours, however, were not common in many international-based reports. For example, several participants told that they had provided volunteering services to some senior citizens and disabled people living alone in rural communities [58]. Having participated in fieldwork and services, they understood that their forces and energies were in demand:

“Many seniors and elderly need us, they need nurses and counsellors … if we do not provide much help to them, they will die … working in the nursing profession should not always be about money … we are there to provide help to our country and communities … I want the government to provide us with additional resources, such as wheelchairs … even if not, we will still help them, no matter what …”

(P#33, Kyushu, Junior)

Another similar message was captured about the long-term patients in the housing facilities [68]:

“Many people in the housing facilities need regular assistance and help from us, such as nurses and counsellors … but during the COVID-19 pandemic, many professionals cannot make their usual visits … I felt so bad because of these missed visits … as nursing students, many of my classmates joined the community centre for the volunteering services … we sent food to the minorities … we visited them, we changed their blankets and masks … As fellow Japanese people, we are here to help …”

(P#16, Chugoku, Sophomore)

#### 3.2.1. Personal Sacrifice: National Developments and Caring Rather Than Personal Interests

Many advocated that being nursing professionals required learning how to sacrifice their own personal interests, goals, and desires because nursing professionals need to take a lot of social responsibility as many people always need their help [19,20,21,22,23,24,25], especially during the COVID-19 pandemic, in which Japan faced lockdown and social distancing as part of the country’s disease protection plan. Particularly, based on the sense of collectivism in Japan [67], Japanese people, including pre-service and in-service public health professionals, usually place the public’s interests and benefits beyond personal goals and development [69]. A previous study indicated that Japanese adolescents are trained and educated to understand the differences between individualism and collectivism from a young age. Japanese people, therefore, should contribute their energies and good for the public interest as their priority [70]. However, due to the shortage of resources, such as food, medicine, masks, and even manpower, some disabled people, long-term patients, and senior citizens [58] could not receive enough support from their regular services of the public health system.

Through the lens of SCCT [19,20,21,22,23,24,25], many participants explained that they had contacted the local public health department and the social welfare department regarding potential volunteering services and coordination. For example, several told that they had worked in a food bank volunteering team to send food to senior citizens and long-term patients in their community:

“Japan has a lot of senior citizens, long-term patients, and an ageing population … therefore, we need to help those people without normal physical condition … this is what we learn from our school. Japanese need to help Japanese … I do not have money, but I have time and power … We formed a group of 81 students, and we did morning and evening teams for the food sending …”

(P#10, Kyushu, Freshman)

Another participant shared that some senior citizens do not have any telephone or cell phone services, and have to enter the neighbourhood in order to check on the situation of other community members:

“Like my grandparents, they don’t have a telephone in their home … but I know a group of community volunteers visit their home for food delivery and caring … of course, we are doing the same thing away from my hometown…but all Japanese people connect with other Japanese people … we are not alone … as long as I sacrifice three hours per day, we have more than a thousand volunteering hours in Japan …”

(P#39, Kanto, Senior)

Besides community visiting, many did some volunteer virtual visiting and counselling with long-term patients and disabled people in housing facilities. In fact, after the minorities received enough food, they demanded mental and psychological support. Due to the social distancing recommendation, through the coordination of a local nonprofit organisation, some participants and their nursing classmates formed online counselling teams as volunteer services:

“Even if we cannot go to the physical housing of the patients and minorities, we can still connect with them with internet and telephone … for sometimes, people want to be touched, connected, and cared for. We still show them that … we care you … We are here as we are Japanese people … we do not have walls between us … I am glad that all our nursing students decided to join this online volunteering service … I called and contacted 20 people regularly as my patients…we shared lived stories and daily activities …”

(P#13, Tohoku, Sophomore)

In short, based on SCCT [19,20,21,22,23,24,25], almost all participants advocated that, without a doubt, the development and care of their country and citizens always play a significant role in their experiences, sense of belonging, and decision-making processes [19,20,21,22,23,24,25]. Based on traditional East Asian perspectives [71,72], it is worth noting that collectivist considerations always inform Japanese people in their daily practices and lives. The COVID-19 pandemic served as the tool to reinforce the participants’ sense of belonging, both as Japanese citizens and as nursing professionals. It is worth noting that a sense of collectivism, benefits for the public interests and contribution to the public communities highly influence how Japanese people understand and describe their career decision-making process and sense of belonging as, in this case, nursing students [70]. These behaviours and senses are significant for Japanese citizens.

#### 3.2.2. Concerning the Need for Future Medical and Nursing Development

Besides the COVID-19 pandemic’s influence and impact on their experiences, sense of belonging, and decision-making processes, a large group of participants advocated that the COVID-19 pandemic is an opportunity for the government and nonprofit organisations to reform and upgrade their current public health system and services [19,20,21,22,23,24,25]. As pre-service nursing professionals and volunteers for some minorities in the community, many advocated that there is room to improve their country [7,61,73]. First, many advocated that the government should establish big data information lists regarding senior citizens, long-term patients, disabled people, and minorities in local communities. During the COVID-19 pandemic, many volunteers had a hard time sending food and locating the right people due to the shortage of information:

“I think the hospital, government, or some agencies should have the location and contact information of people who need care … some senior citizens do not have cell phones or family members with them … fortunately, we have a team of local neighbours and friends in the community … we made our own list for visiting … for food and medicine … this COVID-19 pandemic is a good opportunity for us to upgrade to an internet-based country …”

(P#42, Chubu, Senior)

In fact, although Japan is one of the most developed countries in the East Asian region, many senior citizens, particularly in rural regions [58], do not have cell phones or internet access due to various reasons, such as no internet cable access, monthly fees, and remote locations. Challenging the upper management and the decision of the leadership is not common in Japan due to the sense of collectivism [69]. However, due to the sense of belonging for the public health system, the participants, as volunteers, suggested that the government should upgrade the internet access to all people. Based on SCCT [19,20,21,22,23,24,25], many would like to use their role as nursing professionals to influence the government in reforming and upgrading.

Second, many advocated that there is a need to have a list of telephone counselling services and the matching of counsellors and patients in the community. Currently, Japanese people need to go to public health and community centres for social care services. However, due to the COVID-19 pandemic, many patients refused to leave their home. Therefore, the connections were cut off as the nurses and counsellors could not provide visiting services [12]. Many participants indicated that, as pre-service nursing professionals and Japanese citizens, they would like to use their roles and feedback to influence the development of the public health system [68]. As one participant said:

“nursing is not a single profession … nursing should be connected to other services, such as counselling, social caring, welfare, and even community work … as a nursing student and Japanese citizen, I need to do something to improve the experiences of the patients … I do not understand why professionals cannot go outside their centres for the services … it is time for us to change this behaviour … I am a nurse, I can do that …”

(P#30, Kansai, Junior)

In conclusion, through the lens of SCCT [19,20,21,22,23,24,25], the sharing of the participants revealed how COVID-19 influenced the experiences, sense of belonging, and decision-making processes of a group of Japanese nursing students. Based on two groups of interview questions and protocols, the researcher captured two themes (i.e., sharing about the situation before the COVID-19 pandemic and sharing about the situation during the COVID-19 pandemic). It is worth noting that all participants decided to study nursing as their university major and pursue the nursing profession as their life-long career. More importantly, COVID-19 played an important role in reinforcing their experiences, sense of belonging, and decision-making processes as nursing students and Japanese citizens. Based on the ideas of SCCT [19,20,21,22,23,24,25], and the collectivist behaviours [67,71] of the Japanese tradition, many explained how ideas about patriotism were connected to their sense of belonging and decision-making processes. In this case, the participants showed a strong motivation regarding their nursing student status and confirmed their decision-making process as COVID-19 provided them with informal placements in the community.

## 4. Limitations and Future Research Directions

Every research study has its limitations. Five limitations have been found in this study. First, due to the COVID-19 pandemic and the recommendation of social distancing, the researcher could not conduct any face-to-face interview sessions and focus group activities. Therefore, in-depth information and well-established relationships were not able to be found. However, the researcher overcame these elements by sending a detailed personal and academic background to each, conducting virtual interview sessions, and providing homework tuition and career development suggestions. All these additional steps increased the experiences and relationships between the researcher and participants.

Second, although the COVID-19 pandemic and the recommendations of social distancing limited the face-to-face interview sessions and focus group activities, the researcher has established a set of virtual interview skills and recommendations as the tools for the qualitative research study. This study, however, showed the effectiveness and samples for potential researchers and readers as the blueprint for virtual-based qualitative research studies. Future researchers and scholars may follow the steps for their own research study.

Third, the current study mainly focused on the issues in Japan. However, it is worth noting that other regions, such as China, Taiwan, Hong Kong, Macau, Singapore, Malaysia, Vietnam, and South Korea, may face a similar background and problem. Therefore, future researchers can expand the current research problems and issues to other regions and countries with a similar background for further understanding.

Fourth, as this study was a qualitative research study, no survey and questionnaire were conducted. In order to increase the coverage for a larger population, wider voices and feedback should be collected. However, due to the current COVID-19 pandemic, many pre-service and in-service public health professionals were unwilling to share their experiences, stories and feedback for various reasons. Therefore, future research studies may employ a mixed methodology in order to cover a wider population for a rich and in-depth understanding.

Fifth, the researcher tried to collect a larger population for a quantitative research study. However, due to the limitation of access and the COVID-19 pandemic, only a few individuals were willing to submit the survey for the research study. However, nearly fifty participants were willing to join a qualitative research study. Therefore, future research studies may expand the directions and interests of both mixed and quantitative studies.

## 5. Conclusions

Based on the current academic database, this is one of the very first nursing research studies about the experiences, sense of belonging, and decision-making processes of contemporary nursing students under the influence of the COVID-19 pandemic in Japan. During the interview procedure, the East Asian region was still experiencing the disaster of the COVID-19 pandemic and the social distancing policy. Therefore, many participants were experiencing COVID-19′s influence and described the relationship between COVID-19 and their experiences, sense of belonging, and decision-making processes during the interview sessions.

Through the lens of SCCT, the researcher found that although COVID-19 highly influenced the understanding, feedback, and living standards of the participants, their sense of belonging (i.e., as Japanese citizens and as nursing professionals) and their decision-making processes remained unchanged. In fact, most advocated that the COVID-19 pandemic strongly reinforced their sense of belonging and decision-making processes and reinforced their experiences as pre-service nursing professionals and nursing students as Japanese citizens.

More importantly, based on the connection between SCCT and sense of belonging (i.e., as Japanese citizens), this research study discovered that many Japanese nursing students advocated that Japan’s national development, the benefits and advantages of their country, were of greater importance than their own personal development and goals. Although many had the opportunity to drop and change their nursing major to other educational programmes, most advocated that as Japanese citizens, they had a mission to serve their country during the natural disaster.

## Figures and Tables

**Table 1:** Themes and subthemes.

Themes and Subthemes		
3.1		**Before the COVID-19 Pandemic: I Have the Mission to Help Minorities**
	3.1.1	Promoting Rural and Suburban Public Health Performance and Knowledge
	3.1.2	Increasing the Quality of the Public Health System as a Citizen
3.2		**During the COVID-19 Pandemic: My Country Needs my Effort and Energy**
	3.2.1	Personal Sacrifice: National Developments and Caring Rather than Personal Interests
	3.2.2	Concerning the Need for Medical and Nursing Development

## Appendix A

**Table A1:** Demography of participants.

Name	University Location (Based on Region)	Grade at the University
P#1	Hokkaido	Freshman (1st Year)
P#2	Tohoku	
P#3		
P#4	Kanto	
P#5		
P#6		
P#7	Chubu	
P#8		
P#9	Kyushu	
P#10		
P#11	Tohoku	Sophomore (2nd Year)
P#12		
P#13		
P#14	Kansai	
P#15		
P#16	Chugoku	
P#17		
P#18	Shikoku	
P#19	Kyushu	
P#20		
P#21	Tohoku	Junior (3rd Year)
P#22		
P#23		
P#24	Kanto	
P#25		
P#26		
P#27	Chubu	
P#28		
P#29		
P#30	Kansai	
P#31		
P#32		
P#33	Kyushu	
P#34		
P#35	Hokkaido	Senior (4th Year)
P#36	Tohoku	
P#37		
P#38	Kanto	
P#39		
P#40		
P#41	Chubu	
P#42		
P#43		
P#44	Kansai	
P#45		
P#46	Shikoku	
P#47	Kyushu	
P#48		
P#49	Okinawa	

## Appendix B

Interview question

(1) How would you describe the nursing profession?
(2) How is your nursing student’s experience?
(3) How would you describe your sense of belonging as a nursing student?
(4) Why would you select nursing as your university major?
(5) What make you (i.e., motivations and reasons) want to become a nursing professional in the future or as career development?
(6) How would the COVID-19 pandemic change your life?
(7) How would the COVID-19 pandemic change your experiences as nursing students?
(8) How would the COVID-19 pandemic change your sense of belonging as nursing students and potential nursing professional in the future?
(9) How would the COVID-19 pandemic change your decision-making process as a nursing student and potential nursing professionals in the future?
(10) Any important things (i.e., before the COVID-19 and during the COVID-19 pandemic) you want to tell me?

## References

[1] Cullen L., Titler M.G. (2004). Promoting evidence-based practice: An internship for staff nurses. Worldviews Evid.-Based Nurs..

[2] Jones M. (2017). Career Commitment of Nurse Faculty. Res. Theory Nurs. Pract..

[3] Bandura A., Adams N.E. (1977). Analysis of self-efficacy theory of behavioral change. Cognit. Ther. Res..

[4] Bandura A. (1989). Perceived self-efficacy in the exercise of personal agency. Psychol. Bull. Br. Psychol. Soc..

[5] Bandura A. (1997). Self-Efficacy: The Exercise of Control.

[6] Dos Santos L.M. (2020). I am a nursing student but hate nursing: The East Asian perspectives between social expectation and social context. Int. J. Environ. Res. Public Health.

[7] Dos Santos L.M. (2020). I teach nursing as a male nursing educator: The East Asian perspective, context, and social cognitive career experiences. Int. J. Environ. Res. Public Health.

[8] Ten Hoeve Y., Castelein S., Jansen G., Roodbol P. (2017). Dreams and disappointments regarding nursing: Student nurses’ reasons for attrition and retention. A qualitative study design. Nurse Educ. Today.

[9] Wills N.L., Wilson B., Woodcock E.B., Abraham S.P., Gillum D.R. (2018). Appearance of Nurses and Perceived Professionalism. Int. J. Stud. Nurs..

[10] Yu H.-Y., Tang F.-I., Chen I.-J., Yin T.J.C., Chen C.-C., Yu S. (2016). Nurse administrators’ intentions and considerations in recruiting inactive nurses. J. Nurs. Manag..

[11] Mohamed L.K. (2019). First-career and second-career nurses’ experiences of stress, presenteeism and burnout during transition to practice. Evid. Based Nurs..

[12] Dos Santos L.M. (2020). The challenges of public health, social work, and psychological counselling services in South Korea: The issues of limited support and resource. Int. J. Environ. Res. Public Health.

[13] Adams A., Bond S. (2000). Hospital nurses’ job satisfaction, individual and organisational characteristics. J. Adv. Nurs..

[14] Chang H., Friesner D., Chu T., Huang T., Liao Y., Teng C. (2018). The impact of burnout on self-efficacy, outcome expectations, career interest and nurse turnover. J. Adv. Nurs..

[15] Long S.O. (1986). Roles, careers and femininity in biomedicine: Women physicians and nurses in Japan. Soc. Sci. Med..

[16] Tanaka S., Serizawa T., Sakaguchi C. (2008). Career redevelopment programmes for inactive nurses in Japan. J. Clin. Nurs..

[17] Kubo Y., Hatono Y., Kubo T., Shimamoto S., Nakatani J., Burgel B.J. (2017). Exploring career anchors among occupational health nurses in Japan: A qualitative study. Jpn. J. Nurs. Sci..

[18] Dos Santos L.M., Lo H.F. (2018). The development of doctoral degree curriculum in England: Perspectives from professional doctoral degree graduates. Int. J. Educ. Policy Leadersh..

[19] Swanson J., Gore P., Brown S.D., Lent R.W. (2000). Advances in vocational psychology theory and research. Handbook of counseling Psychology.

[20] Lent R.W., Brown S.D., Brenner B., Chopra S., Davis T., Talleyrand R., Suthakaran V. (2001). The role of contextual supports and barriers in the choice of math/science educational options: A test of social cognitive hypotheses. J. Couns. Psychol..

[21] Lent R.W., Brown S.D. (2008). Social cognitive career theory and subjective well-being in the context of work. J. Career Assess..

[22] Brown S.D., Lent R.W. (2017). Social cognitive career theory in a diverse world. J. Career Assess..

[23] Lent R.W., Brown S.D., Hackett G. (2000). Contextual supports and barriers to career choice: A social cognitive analysis. J. Couns. Psychol..

[24] Lent R.W., Brown S.D., Hackett G. (1994). Toward a unifying social cognitive theory of career and academic interest, choice, and performance. J. Vocat. Behav..

[25] Lent R.W., Brown S.D. (1996). Social cognitive approach to career development: An overview. Career Dev. Q..

[26] Dos Santos L.M. (2019). Pre-service teachers’ professional development through four-step problem-solving model: A seminar method. Int. J. Educ. Pract..

[27] Dos Santos L.M. (2018). Career decision of recent first-generation postsecondary graduates at a metropolitan region in Canada: A social cognitive career theory approach. Alta. J. Educ. Res..

[28] Dos Santos L.M. (2019). Rural Public Health Workforce Training and Development: The Performance of an Undergraduate Internship Programme in a Rural Hospital and Healthcare Centre. Int. J. Environ. Res. Public Health.

[29] Dos Santos L.M. (2019). Engineering education as a second career: The experience of female practising engineers. Glob. J. Eng. Educ..

[30] Brown S.D., Lent R.W. (2019). Social cognitive career theory at 25: Progress in studying the domain satisfaction and career self-management models. J. Career Assess..

[31] Bandura A. (1991). Human agency: The rhetoric and the reality. Am. Psychol..

[32] Bandura A. (1989). Regulation of cognitive processes through perceived self-efficacy. Dev. Psychol..

[33] Dos Santos L.M. (2019). Recruitment and retention of international school teachers in remote archipelagic countries: The Fiji experience. Educ. Sci..

[34] Tanaka S., Maruyama Y., Ooshima S., Ito H. (2011). Working condition of nurses in Japan: Awareness of work-life balance among nursing personnel at a university hospital. J. Clin. Nurs..

[35] Tang K.H., Dos Santos L.M. (2017). A brief discussion and application of interpretative phenomenological analysis in the field of health science and public health. Int. J. Learn. Dev..

[36] Dos Santos L.M. (2019). English language learning for engineering students: Application of a visual-only video teaching strategy. Glob. J. Eng. Educ..

[37] Dos Santos L.M. (2020). Promoting safer sexual behaviours by employing social cognitive theory among gay university students: A pilot study of a peer modelling programme. Int. J. Environ. Res. Public Health.

[38] Sharan B., Merriam E.J.T. (2015). Qualitative Research: A Guide to Design and Implementation.

[39] Creswell J. (2014). Research Design: Qualitative, Quantitative, and Mixed Methods Appraoches.

[40] Connelly F.M., Clandinin D.J. (1990). Stories of experience and narrative inquiry. Educ. Res..

[41] Clandnin D., Connelly F. (2000). Narrative Inquiry: Experience and Story in Qualitative Research.

[42] Dos Santos L.M. (2019). Experiences and expectations of international students at historically black colleges and universities: An interpretative phenomenological analysis. Educ. Sci..

[43] Dos Santos L.M. (2020). Stress, burnout, and turnover issues of Black expatriate education professionals in South Korea: Social biases, discrimination, and workplace bullying. Int. J. Environ. Res. Public Health.

[44] Dos Santos L.M. (2020). The motivation and experience of distance learning engineering programmes students: A study of non-traditional, returning, evening, and adult students. Int. J. Educ. Pract..

[45] Number of Schools and Student Capacity by Year.

[46] Merriam S.B. (2009). Qualitative Research: A Guide to Design and Implementation.

[47] Moustakas C. (1994). Phenomenological Research Methods.

[48] Thomas D.R. (2006). A general inductive approach for analysing qualitative evaluation data. Am. J. Eval..

[49] Seidman I. (2006). Interviewing as Qualitative Research: A Guide for Researchers in Education and the Social Sciences.

[50] Seidman I. (2013). Interviewing as Qualitative Research: A Guide for Researchers in Education and the Social Sciences.

[51] Robson C. (2002). Real World Research: A Resource for Social Scientists and Practitioner-Researchers.

[52] Marshall B. (2018). Learning to Be Modern: Japanese Political Discourse on Education.

[53] Kippenbrock T. (1990). School of Nursing Variables Related to Male Student College Choice. J. Nurs. Educ..

[54] Pathman D.E., Konrad T.R., Dann R., Koch G. (2004). Retention of primary care physicians in rural health professional shortage areas. Am. J. Public Health.

[55] Bandura A. (1988). Self-efficacy conception of anxiety. Anxiety Res..

[56] Bandura A. (1995). Self-Efficacy in Changing Societies.

[57] Betoret F. (2009). Self-efficacy, school resources, job stressors and burnout among Spanish primary and secondary school teachers: A structural equation approach. Educ. Psychol..

[58] Murayama Y., Inoue K., Yamazaki C., Kameo S., Nakazawa M., Koyama H. (2019). Association between depressive state and lifestyle factors among residents in a rural area in Japan: A cross-sectional study. Tohoku J. Exp. Med..

[59] Matsushima M., Shimizutani S., Yamada H. (2018). Life course consequences of low birth weight: Evidence from Japan. J. Jpn. Int. Econ..

[60] Miyata M., Toyoshima K., Yoda H., Murase M., Kawato H., Yamamoto K., Tanaka K., Kotani M., Kobayashi M. (2016). Extensive use of vasodilator agents and functional echocardiography to monitor extremely-low-birth-weight infants in Japan. J. Neonatal. Perinatal. Med..

[61] Dabney B.W., Linton M., Koonmen J. (2017). School nurses and RN to BSN nursing students. NASN Sch. Nurse.

[62] Copeland D., Harbaugh B.L. (2005). Differences in parenting stress between married and single first time mothers at six to eight weeks after birth. Issues Compr. Pediatr. Nurs..

[63] Rainbow J.G., Steege L.M. (2019). Transition to practice experiences of first- and second-career nurses: A mixed-methods study. J. Clin. Nurs..

[64] Tzeng H.-M. (2002). The influence of nurses’ working motivation and job satisfaction on intention to quit: An empirical investigation in Taiwan. Int. J. Nurs. Stud..

[65] Dos Santos L.M. (2017). The relationship between personal beliefs and teaching practice of ESL teachers at an Asian community centre in Vancouver: A qualitative research in progress. Alta. J. Educ. Res..

[66] Dos Santos L.M. (2018). Postgraduate international students’ living and learning experience at a public university in British Columbia. Alta. J. Educ. Res..

[67] Campion L.L., Wang C.X. (2019). Collectivism and Individualism: The Differentiation of Leadership. TechTrends.

[68] Sugimoto K., Ogata Y., Kashiwagi M., Ueno H., Yumoto Y., Yonekura Y. (2017). Factors associated with deaths in ‘Elderly Housing with Care Services’ in Japan: A cross-sectional study. BMC Palliat. Care.

[69] Nakagawa S., Takeuchi H., Taki Y., Nouchi R., Kotozaki Y., Shinada T., Maruyama T., Sekiguchi A., Iizuka K., Yokoyama R. (2019). Mean diffusivity related to collectivism among university students in Japan. Sci. Rep..

[70] Sugimura K. (2020). Adolescent identity development in Japan. Child Dev. Perspect..

[71] Triandis H.C. (1995). Individualism and Collectivism.

[72] Han C.M. (2017). Individualism, collectivism, and consumer animosity in emerging Asia: Evidence from Korea. J. Consum. Mark..

[73] Del Prato D.M. (2017). Transforming Nursing Education: Fostering Student Development towards Self-Authorship. Int. J. Nurs. Educ. Scholarsh..

